# Doctors’ Mistakes and Blunders

**Published:** 1881-06

**Authors:** 


Doctors' Mistakes and Blunders.—Seeing that so few of your host of subscribers were willing to throw their colors to the breeze and come up like true Spartans and give us a piece of a column now and then, on that very interesting subject, "Doctors' Blunders," I hasten to contribute my mite, deeming it a duty of every physician, which he owes his medical brethren, particularly the novices, to publish his failures as well as his successes But possibly all who have ever sinned have confessed, have been heard from in the column of "Blunders;" nevertheless, my conviction is, if every case of the kind came to light, where the physician saved his neck, so to speak, by adroitly covering in his tracks, they would fill a book larger than Webster's Dictionary.

Why does not some ambitious book writer come to the front with a treatise on this subject? It would outstrip any work in the book market, in the way of sales, and would immortalize his name besides.

Of course its sales should be limited to the medical fraternity, else it might place us in a bad light. No doubt it would at first seem a curiosity; but then we must move early, with our eyes wide open, to be up with the enterprise of the nineteenth century.

Let us have the cases of pneumonia and other diseases which have been strangled by paralyzing doses of poison—veratrum, aconite, belladonna, digitalis, strychnia and morphia! Those which have come to an untimely end by the injudicious use of the lancet and leech; those cases wherein we are told the way to strengthen is to weaken.

Let us hear from those health officers who are sending cases of scarlatina, measles, chicken pox and nettle rash to the pest-house, that they may be treated for smallpox.

A case was reported in New York city some time since, of a youth who was spirited away from his father's comfortable dwelling, amid fumes of burning sulphur, in a closed van, to the Island Hospital. There the doctors declared he had measles. He was detained long enough to absorb sufficient of the variolous contagion to infect a whole neighborhood, however. Thousands of mistakes (*errors in diagnosis?*) might be found.

I shall delay no longer with preliminaries, but launch out and give the history of one among the many of my own. About the middle of the summer of 1880, a young man with his wife came to my office for the purpose of securing my services when she would be confined, saying she expected it would be in one month from that time. She was very anxious to learn as to whether I could be found in at night or not, and so on. Shortly they both left. The month passed, another came and went, but no Mr. B. called. Alas! no call to Mrs. B., I said to myself; ten dollars out. My patient could certainly not have held out so long; and to confirm my suspicions I soon met Mr. B. in the street, but, to avoid me, he crossed to another corner. But, thanks to my lucky planet, bang! goes the gong. I raise the window, Mr. B. is there, and in a hurried and anxious voice calls me to go and see his wife, who is *sick*, and to bring such things as I might need. Well, it did not take long for me to get into my clothes. Let us see what I need. Ergot, pernitrate of iron, iodine, opium, tape, and a good, stiff-backed syringe. Now, with a copy of Handy Andy crammed into my pocket, away I ran, soon reaching the sick chamber, nearly out of breath, fearing that I might arrive in time to be too late. I made all possible haste. Off went my hat and coat, and into the sick chamber I went. Mrs. B. had so much pain that she could not answer me, whether the pain was in her back or bowels, or, in fact, anything. Not being able to make her yield to an examination, and the pains ceasing entirely—as the obstetric teacher tells us, when the doctor arrives, they often do—I made her swallow a good full drachm of the fld. extract of ergot (Squibb's), quickly followed by a cup of ginger tea. Now, says I, comfortably stretching myself in a large, soft rocking chair, I will read a while. O, Doctor, she cries, and up I jumped; the pain, she said, would kill her; the medicine is doing the work, I said. She turned on her side, declaring I knew nothing; a whim too trifling to notice. I told her she would think differently by and by. I made my way as well as I could, up to the mons veneris. Vagina moist; natural heat. The os uteri patulous, readily admitting the finger.

The presentation and bag of waters is what I am after. As I kept my finger there for a moment, I rapidly revolved in my mind the different presentations and possible monsters, though sharply eyed by the nurse and savagely stared at by the husband, from another room. Still I could not forget what I once heard the late lamented Dr. Chas. A. Budd say, in his lectures: "That sometimes one could not tell, in making a vaginal examination, whether he was afoot or on horseback." While in this momentary quandary, I heard something that made my hair stand from the very roots, and my knees suddenly weaken—my heart seemed packed into my trachea—the cry of an infant in a cradle, covered with a canopy! There! get out, you quack, shouted the patient, the lying in? I said you knew nothing. The husband swore he would prosecute, and I got out with my life, but no fee.—Thos. H. Manley, M D. in *The Medical and Surgical Reporter*.

